# Exosomal Non-Coding RNA Mediates Macrophage Polarization: Roles in Cardiovascular Diseases

**DOI:** 10.3390/biology12050745

**Published:** 2023-05-19

**Authors:** Hongyun Wang, Xuan Ye, Michail Spanos, Huanxin Wang, Zijiang Yang, Guoping Li, Junjie Xiao, Lei Zhou

**Affiliations:** 1 Cardiac Regeneration and Ageing Lab, Institute of Cardiovascular Sciences, Shanghai Engineering Research Center of Organ Repair, School of Life Science, Shanghai University, 333 Nan Chen Road, Shanghai 200444, China; 2 Institute of Geriatrics, Affiliated Nantong Hospital of Shanghai University (The Sixth People’s Hospital of Nantong), School of Medicine, Shanghai University, Nantong 226011, China; 3 Department of Cardiology, First Affiliated Hospital of Nanjing Medical University, Nanjing 210008, China; 4 Division of Cardiology, Massachusetts General Hospital and Harvard Medical School, Boston, MA 02114, USA

**Keywords:** extracellular vesicles, non-coding RNA, macrophage polarization, cardiovascular diseases

## Abstract

**Simple Summary:**

The role of exosomal non-coding RNA (ncRNA) in regulating macrophage polarization and the role of polarized macrophages as an important source of extracellular vesicles in cardiovascular diseases remains to be elucidated. In this review, we aim to summarize the role and molecular mechanisms of exosomal-ncRNA in regulating macrophage polarization and contributing to cardiovascular diseases. We also discuss the role of polarized macrophages and their derived extracellular vesicles as well as the therapeutic prospects of exosomal ncRNA in the treatment of cardiovascular diseases.

**Abstract:**

Extracellular vesicles (EVs) or exosomes are nanosized extracellular particles that contain proteins, DNA, non-coding RNA (ncRNA) and other molecules, which are widely present in biofluids throughout the body. As a key mediator of intercellular communication, EVs transfer their cargoes to target cells and activate signaling transduction. Increasing evidence shows that ncRNA is involved in a variety of pathological and physiological processes through various pathways, particularly the inflammatory response. Macrophage, one of the body’s “gatekeepers”, plays a crucial role in inflammatory reactions. Generally, macrophages can be classified as pro-inflammatory type (M1) or anti-inflammatory type (M2) upon their phenotypes, a phenomenon termed macrophage polarization. Increasing evidence indicates that the polarization of macrophages plays important roles in the progression of cardiovascular diseases (CVD). However, the role of exosomal ncRNA in regulating macrophage polarization and the role of polarized macrophages as an important source of EV in CVD remains to be elucidated. In this review, we summarize the role and molecular mechanisms of exosomal-ncRNA in regulating macrophage polarization during CVD development, focusing on their cellular origins, functional cargo, and their detailed effects on macrophage polarization. We also discuss the role of polarized macrophages and their derived EV in CVD as well as the therapeutic prospects of exosomal ncRNA in the treatment of CVD.

## 1. Introduction

Globally, cardiovascular disease (CVD) has become the leading cause of mortality among non-communicable diseases. Over the period 1999–2018, there were 80 million live births and 238 deaths as a result of CVD, which indicates a significant increase in pregnancy-associated mortality rates in the United States [1]. It has been reported that CVD causes more than four million deaths in Europe each year, accounting for 45% of all fatalities [2]. Among all the cardiovascular disorders, atherosclerotic CVD, such as ischemic heart disease (IHD) is a growing burden on Chinese society [3]. Notably, the present data do not include the large number of individuals who did not attend hospitals or who were not treated for diabetes or hypertension [4]. Even though rapid advances have been made in the treatment of CVD, cardiac dysfunction remains a challenge. Exploring new effective therapeutical strategies to treat CVD is imperative [5].

Extracellular vesicles (EVs), secreted by almost every type of cell, are widely present in biofluids such as blood, urine, and tears [6,7,8]. According to their biogenesis and size, EVs can be classified as exosomes, microvesicles, and apoptotic bodies, however, the guidelines for minimal information for studies of EVs 2018 (MISEV18) commonly use the generic term EVs [9]. Increasing evidence has demonstrated that EVs mediate cell to cell communication by delivering biological molecules such as RNA, lipids and proteins [10,11,12] and that they can regulate cell proliferation and immune response [13,14]. Notably, the development of cardiovascular dysfunction involves several kinds of cells including endothelial cells, mesenchymal stem cells, and adipocytes, whose inter-communication plays an important role in regulating information transfer.

Only 2% of transcribed RNAs encode proteins. The rest do not possess translative potential since they lack protein-coding regions (non-coding RNA, ncRNA), including long non-coding RNA (lncRNA), microRNA (miRNA), and circular RNA (circRNA) [15].ncRNAs contribute to a number of pathophysiological processes, such as cell proliferation, differentiation, and apoptosis through regulating gene expression at the transcriptional and post-transcriptional levels. Numerous studies have revealed that ncRNA plays an important role in the development, diagnosis, and treatment of CVD [16,17]. Increasing evidence shows that ncRNA functions as cargo encapsulated by EVs (termed exosomal ncRNA) and then transported stably avoiding degradation [18]. A deeper understanding of the role and mechanisms underlying exosomal ncRNAs in the regulation of CVD will enhance our understanding of the diagnosis and treatment of them.

Among immunocytes, macrophages play a critical role during both intrinsic and adaptive immunity, participating in numerous pathophysiological processes including inflammation and tissue repair following injury. In general, macrophages are capable of exhibiting distinct functional differences in response to different stimuli, both in vivo and in vitro. Macrophages are classically divided into two subtypes based on their active status and functions, namely M1 type (activated macrophage) and M2 type (alternatively activated macrophage) [19]. Macrophages are polarized into M1 when stimulated by lipopolysaccharide (LPS) or pro-inflammatory cytokines and secret inflammatory factors as a result. M1 macrophages are involved in early-stage inflammatory reactions through producing pro-inflammatory factors including interleukin-1-β (IL-1β), IL-6, IL-12 and tumor necrosis factor-α (TNF-α), with phenotypically high expression of MHC-II, CD68, CD80, and CD86. In contrast to M1 polarization, M2 macrophages are induced by cytokines such as IL-4, IL-13, and secret anti-inflammatory factors (TGF-β, IL-1RA, and IL-10), which are involved in tissue repair, wound healing, and angiogenesis [20,21,22].

Recent studies suggest that EVs are involved in macrophage polarization in response to different microenvironments, thus contributing to pathological processes such as inflammation, lipid metabolism, and endothelial damage, and therefore play an important role in CVD such as ischemia-reperfusion injury (IRI), atherosclerosis (AS) and myocardial infarction (MI). The goal of this review is to examine the mechanisms that enable EV-enclosed ncRNA to regulate macrophage polarization and their detailed impacts on macrophages. In addition, we will discuss the effect of EVs on CVD and potential treatments.

## 2. Molecular Mechanism of Exosomal ncRNA Regulating Macrophage Polarization

EVs are considered important “messenger” of intercellular communication. Due to their natural molecule cargo, EVs are capable of transmitting critical information and triggering parallel signaling pathways in target cells. Uptake of donor EV-enclosed ncRNA by recipient cells is important for the communication between different cells or organs. Evidence suggests that the EV-enclosed ncRNA can complementarily bind to the untranslated region at the 3′ end of the mRNA, leading to a reduced translation, which results in pathophysiological changes in the recipient [23,24].

MiRNA is the most abundant class of ncRNA in EVs, averaging 22 nucleotides in length, and plays a critical role in regulating post-transcriptional of gene expression by targeting mRNA [25,26,27]. A number of miRs have been identified as being involved in macrophage polarization, especially within mesenchymal stem cells (MSC)-derived EVs. MiR-223 is an anti-inflammatory miRNA that regulates macrophage polarization, inhibits macrophage activation, promotes the expression of macrophage markers associated with M2 cells, and enhances the anti-inflammatory response by modulating the nuclear factor kappa-B signaling pathway and signal transducers and activators of transduction-3 (STAT3) pathways [28,29]. Interestingly, MSC-derived EV-enclosed miR-223 was shown to target PBX/knotted 1 homeobox 1 (Pknox1) to regulate macrophage polarization) [30]. In addition, MSC-derived EV-enclosed miR-21a-5p promotes M2 polarization by suppressing the expression of Krüppel-like factor-6 (KLF6) and reduces the expression of M1 macrophage markers (IL-1β, IL-6, and TNFα), thereby mitigating inflammatory response [31,32]. Tension homolog deleted on chromosome ten (PTEN) is also inhibited by miR-21-5p causing the M2 polarization of macrophages [33]. EV-contained miR-21-5p has different effects depending on its cellular origin, which can induce anti-inflammatory or inflammatory reactions by activating different signaling pathways in the reception cells. Chen et al. isolated EVs from neonatal mouse cardiomyocytes and used them to treat macrophages examining the state of macrophage polarization. The enriched EV-miR-146a-5p promoted the expression of vascular endothelial growth factor-A (VEGFA) and inhibited the expression of TNFα or inducible nitric oxide synthase (iNOS) [34].

There is considerable evidence that circular RNA (circRNA), a well-studied class of ncRNA, can regulate pre-/post-transcription by sponging miRNA and coding proteins, which play crucial roles in the polarization of macrophages [35]. Yang et al. found that circ-Rps5 transmitted by hypoxic-pretreated adipose stem cell-derived EVs could promote microglia/macrophage M2 polarization by targeting silent regulatory protein-7 (SIRT7) and miR-124-3p [36].

A long-nucleotide RNA molecule (lncRNA) is a class of functional RNA molecules larger than 200 nucleotides that do not encode proteins and participate in gene transcription, translation, and post-translational modification [37]. Accumulating studies have shown that lncRNA can act as sponges for miRNA or as decoys for transcription factors by interfering with specific signaling pathways to promote macrophage phenotype transformation [38]. Ma et al. demonstrate that endothelial progenitor cell-derived EVs suppress the down-regulation effect of miR-9-5p on SIRT1 and promote the polarization of M2 macrophage through the transfer of lncRNA TUG1 [39]. It has been found that angiotensin-II (Ang-II) treated cardiomyocytes are capable of delivering plasmacytoma variant translocation-1 (PVT1) via secreting EVs, and they are also able to regulate the levels of miR-145-5p and interleukin-16 [40]. Plasma EV-lnc-MRGPRF-6:1 upregulation promotes macrophage M1 polarization and inflammation in coronary atherosclerosis patients by activating toll-like receptor-4, myeloid differentiation factor-88, and mitogen-activated protein kinases (TLR4-MyD88-MAPK) [41]. In acute pancreatitis, Metastasis-associated lung adenocarcinoma transcript-1 (MALAT1) embedded in EVs competitively binds to miR-181a-5p and induces M1 macrophages, further regulating high mobility group box protein-1 (HMGB1)-TLR4 signaling pathway, thereby upregulating TNF-α and IL-6 levels [42]. In contrast, while exosomal MALAT1 is also elevated when human vein endothelial cells are treated with oxLDL (oxidized low-density lipoprotein), however, in that setting it promotes macrophage polarization into M2 [43].

Overall, these studies demonstrate that ncRNA, which is an important component of EV cargo, is closely associated with macrophage polarization and is extensively involved in regulating inflammatory or anti-inflammatory signaling pathways.

## 3. Role of Exosomal ncRNA in CVD-Associated Macrophage Polarization

Increasing evidence shows that macrophage activation can induce the secretion of IL-18 and IL-1β, both of which can exacerbate CVD development and its complications. In this section, we summarize the role of EV-contained ncRNA in regulating macrophage polarization that contributes to CVD.

### 3.1. Atherosclerosis

**Figure 1:**
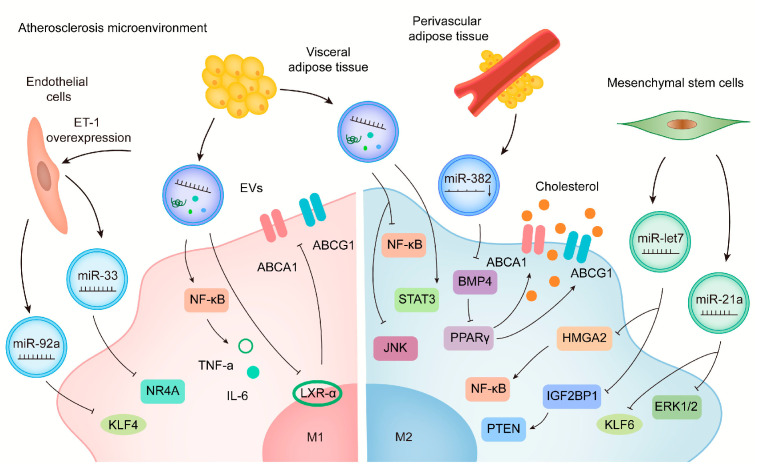
Mechanisms involved in the regulation of macrophage polarization by exosomal ncRNA in AS. Exosomes originating from a variety of cells or tissues can target macrophages by transmitting ncRNA and macrophages can polarize into different functional phenotypes. ECs secrete miR-92a-rich exosomes, which can promote macrophage to polarize toward the M1 type by targeting KLF4. With endothelial-specific overexpression of ET-1, the exosomal miR-33 directly targets NR4A. The exosomes isolated from VAT of obese mice can be absorbed by macrophages and promote the production of macrophage foam cells by down-regulating the cholesterol efflux mediated by ABCA1 and ABCG1. It can induce M1 phenotype change and pro-inflammatory cytokines TNF-a and IL-6 secretion in macrophages, accompanied by increased phosphorylation of NF-κB and a decreased cellular level of LXR-α. EVs derived from adipose tissue mesenchymal stem cells can decrease LPS-induced M1 expression by inhibiting JNK and NF-κB pathways, and increase M2 expression by activating the STAT3 pathway. MiR-let7 family is highly enriched in MSC exosomes, which promotes macrophage M2 polarization through HMGA2/NF-κB pathway and inhibits macrophage infiltration in plaques through IGF2BP1/PTEN pathway. MSCs exosomal miR-21a-5p can promote macrophage M2 polarization by targeting KLF6 and suppress macrophage migration by inhibiting the ERK1/2 signaling pathway.

Atherosclerosis (AS), an inflammatory disease caused by lipid deposition resulting in damage to the vascular walls, is characterized primarily by the transformation of macrophages into foam cells [7,44]. EVs originating from a variety of tissues or cells can transmit ncRNA to macrophages, thereby regulating their polarization (Figure 1) and affecting inflammation, lipid metabolism, and other pathological processes associated with AS.

In addition to their basic structural component, endothelial cells (ECs) play a critical role in the progression and development of atherosclerosis. In an atherosclerosis-prone microenvironment, ECs secrete miR-92a-rich EVs that promote macrophage polarization toward the M1 type and enhance their pro-inflammatory capacity. Previous experiments demonstrated that miR-92a regulates macrophage polarization by targeting Krüppel-like factors (KLF4) [45]. Apolipoprotein E (ApoE) knockout mice with endothelial-specific overexpression of endothelin-1 (ET-1) exhibit a significant increase in atherosclerotic plaque size along with increased pro-inflammatory cytokine secretion. The ET-1-enriched EVs derived from human vein endothelial cells can promote the expression of M1-related genes while decreasing the expression of M2-related genes. Bioinformatics analysis indicates that miR-33 directly targets orphan nuclear receptor-4-A (NR4A) and promotes the activation of pro-inflammatory macrophages, which can be reversed by knocking out ET-1. Taken together, these data suggest that EV-enclosed ET-1 may be a potential therapeutic option for treating AS [46]. The related mechanism of ECs in regulating macrophage polarization is also shown in Figure 1.

As obesity is one of the major causes of AS, EVs from adipose tissue (AT) play a significant role in obesity-associated metabolic complications as well as the progression of AS. EVs derived from visceral adipose tissue (VAT) can be absorbed by macrophages and promote foam cell production by reducing ABCA1-mediated cholesterol efflux in obese mice exposed to high-fat diets (HFD). VAT-EVs can also induce the secretion of TNF-a and IL-6, thereby inducing M1 phenotype, accompanied by increased phosphorylation of NF-κB. Intravenously administration of HFD-VAT-derived EVs significantly exacerbates the development of AS in ApoE^−/−^ mice [47]. In addition, perivascular adipose tissue can release EVs (PVAT-EXO) to regulate inflammation and vascular homeostasis. According to Liu et al., patients with coronary heart disease (CHD) have lower levels of miR-382-5p content in PVAT-EXO. MiR-382-5p reduces the uptake of cholesterol by macrophages as well as the formation of foam cells via up-regulating the content of ATP-binding cassette transporter-A1 (ABCA1) and peroxisome proliferators-activated receptor-γ (BMP4-PPARγ) pathway [48]. MSC-derived EVs from adipose tissue reduce lipopolysaccharide (LPS)-induced inflammatory M1-related markers while increasing the expression of M2-related makers through the STAT3 pathway [49]. According to these studies, there may be a new crosstalk between adipose tissue and macrophages that contributes to obesity-related AS. In addition to immune regulation and immunosuppression, EVs derived from MSCs are also involved in cardiac repair. In fact, the regulation of macrophage polarization by EV-enclosed miRNA is considered to be an important mechanism for cardiac protection. In atherosclerotic mice, MSC-derived EVs reduce the infiltration of macrophages by polarizing macrophages to the M2 type. In particular, MSC-derived EVs containing miR-let7 (EV-miR-let7) are capable of mitigating the development of AS. EV-miR-let7 can induce M2 polarization and attenuate macrophage infiltration in plaques through IGF2BP1/PTEN pathway [50] (Figure 1). Similarly, MSC-derived EV-miR-21a-5p promotes M2 polarization of macrophages and suppresses macrophage migration through targeting KLF6 and signal-regulated-kinase-1/2 (ERK1/2), which ultimately alleviates the development of AS [31]. Studies such as these provide new insight into the mechanism of action of MSC-derived EV-ncRNA on macrophages as well as the pathological process of AS, thereby providing a promising therapeutic method for reducing coronary artery disease risks. Recently, EV-based gene therapies or engineering EVs have emerged as a potential treatment for CVD. Engineered EVs containing anti-miR-33a-5p are constructed to increase the expression of anti-miR-33a-5p in endothelial cells. When EV-enclosed miR-33a-5p is released from ECs, it increases cholesterol efflux from macrophages and smooth muscle cells, which will slow down the pathological process of AS [51].

### 3.2. Myocardial Infarction and Ischemia-Reperfusion Injury

Myocardial infarction (MI), caused by a sudden interruption of coronary blood flow, has become the leading risk for CVD-associated mortality. Reperfusion therapy is considered the common approach to reducing the infarct size post MI, however, it also induces myocardial ischemia-reperfusion injury (IRI) leading to cascade reactions of cardiac inflammation [52,53]. Accumulating studies have demonstrated that EV cargo can transmit cardioprotective molecules to the myocardium and activate the PTEN/AKT pathway, which attenuates the apoptosis induced by IRI in cardiomyocytes [6]. As a result of IRI, EVs are released in the heart, which leads to M1 polarization of macrophages, increased expression of inflammatory cytokines, and even distant organ inflammation [54]. Zhang et al. showed that EVs derived from H9C2 cells (ExoQuick Precipitation, at a dose of 45 μg/μL, 48 h) subjected to hypoxia promote M2 macrophage polarization and reduce cardiomyocyte apoptosis [55]. However, another study provided a contrary result using differential centrifugation-extracted EVs. EVs released by cardiomyocytes under ischemic conditions have been shown to stimulate inflammatory factor expression through p38 MAPK signaling, enhancing macrophage adhesion to fibronectin, and thereby affecting the repair of injuries and remodeling of myocardium after infarction [56]. Three possible reasons may contribute to this contrary, which includes extraction approach differences, usage doze variation, and treatment time of EVs. Therefore, it should consider these factors when investigating the role of EVs in mediating cross-talk between cardiomyocytes and macrophages.

Notably, the interaction between macrophage polarization and fibroblasts also plays an important role in contributing to MI and IRI. For example, macrophage-derived exosomal miR-155 inhibited the proliferation of fibroblast, thereby regulating collagen production post-MI [57]. Reducing M2 macrophages produced exosome-enclosed lncRNA-ASLNCS5088 could improve macrophage-related fibroblast activation [58]. It should be noted that several pieces of evidence showed that several cross-talk between fibroblasts and macrophages was independent of exosome or extracellular vesicles, which was not our present focus.

Stem cells have been widely studied for their repair and regeneration effects on tissues, and their role in CVD is receiving increasing attention. EVs produced by MSCs, cardio-sphere derived cells (CDCs), or cardiac progenitor cells (CPCs) are considered alternatives to stem cell-based therapy, providing a new direction for cell-free nano-therapy for MI and IRI.

**Figure 2:**
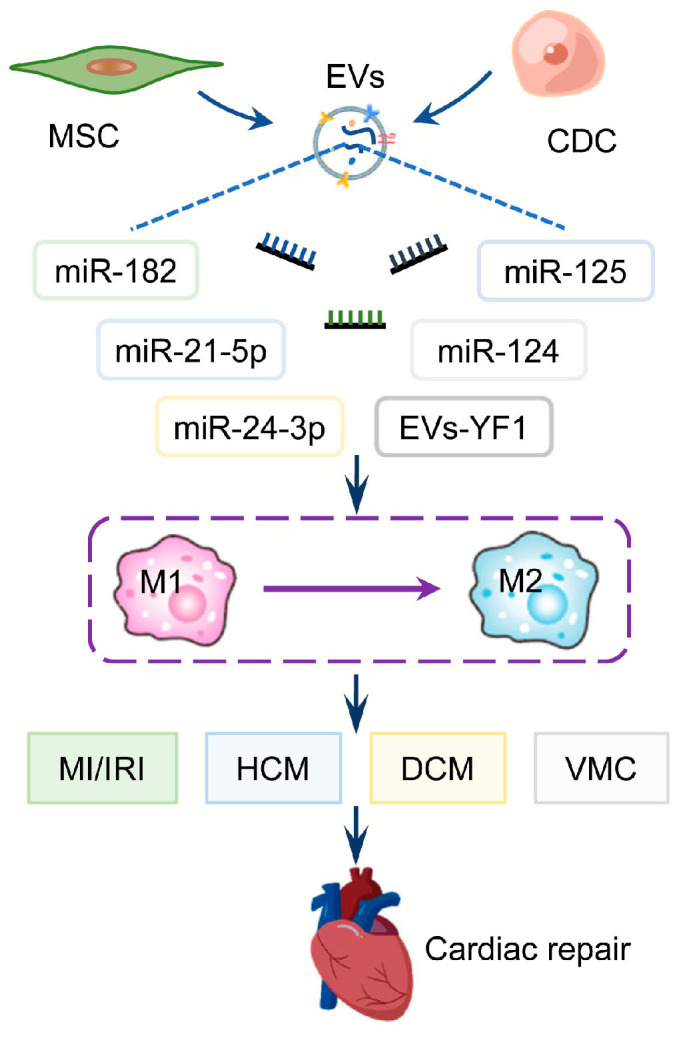
Macrophage polarization associated therapeutical EVs-ncRNAs in CVD. MSC or CDC-derived EVs regulate the transition from M1 to M2 polarization of macrophages by delivering ncRNAs such as miR-182, miR-21-5p, miR-24-3p, miR-124, miR-125, and YF1. These EVs-ncRNAs can protect the heart against MI/IRI, HCM, DCM, and VMC by regulating macrophage polarization. Abbreviations: MSC: mesenchymal stem cell; CDC: cardio-sphere derived cell; EVs: extracellular vesicles; YF1: Y RNA fragment 1; MI/IRI: myocardial infarction/ischemia-reperfusion injury; HCM: hypertrophic cardiomyopathy; DCM: diabetic cardiomyopathy; VMC: viral myocarditis.

Injecting MSC-derived EVs into the myocardium reduces the infarct size as well as serum inflammatory levels (Figure 2). In addition, EVs mediate the transition from M1 polarization to M2 polarization of macrophages via regulating miR-182 and its downstream targets [59]. The intravenous administration of MSC-EVs significantly reduces the expression of M1 markers while increasing M2 markers, thereby reducing the inflammatory response. In vitro studies show that EVs promote macrophage polarization towards the M2 phenotype by delivering miR-21-5p which reduces inflammation and promotes cardiac repair [32]. EVs extracted from adipose-derived MSCs (ADSC) attenuate myocardial injury, myocardial apoptosis, myocardial fibrosis, and inflammatory response after MI by promoting macrophage M2 polarization through activating sphingosine-1-phosphate/sphingosine kinase 1/sphingosine-1-phosphate receptor 1 (S1P/SK1/S1PR1) signaling pathway [60]. According to Zhu et al., EV-miR-24-3p derived from human umbilical cord MSCs promotes M2 macrophage polarization by inhibiting phospholipase C/beta 3 (Plcb3) expression and improves cardiac function after MI [61]. A combination of MSCs-derived EVs with alginate gel can increase the cardiac retention of EVs and enhance the efficiency of EVs treatment. As a result, cardiomyocyte apoptosis is attenuated in the infarct border area and macrophage polarization to M2 is enhanced [62]. EVs derived from low concentrations of LPS-pretreated MSCs protect against LPS injury, which opens up a new horizon in cell-free therapy [63]. Fibronectin type III domain-containing protein-5 (FNDC5) is a transmembrane protein that plays an important role in the repair of inflammatory diseases including MI. MSCs pre-treated with FNDC5 release EVs (FNDC5-MSCs-EXO) that exhibit superior anti-inflammatory, anti-apoptotic, and M2 macrophage polarizing effects via activating the Nrf2/HO-1 axis [64]. A co-culture experiment of MSCs and macrophages confirms that EVs secreted by MSCs promote wound healing and macrophage M2 phenotypic transition by regulating miR-124 and miR-125 levels [65]. Loading miR-101a into MSCs-derived EVs can reduce myocardial infarct size and improve ejection fraction and shortening fraction [66].

The cardioprotective effect of CDCs is partly mediated by paracrine secretion of EVs, whose production can polarize M1 macrophages to a proangiogenic phenotype by upregulating arginase 1, ultimately reducing nitric oxide secretion in activated macrophages [67]. Intramyocardial or intracoronary injection of CDCs-derived EVs can reduce the level of inflammatory cytokines, increase the level of IL-10 and minimize the myocardial infarction area in rats and pigs. One of the most abundant RNA fragments enclosed in CDC-derived EVs, Y RNA fragment 1 (EVs-YF1), stimulates IL-10 transcription and secretion from macrophages and protects cardiomyocytes from oxidative stress. In fact, intracoronary injection of EVs-YF1 after MIR was shown to reduce myocardial infarct area [68] (Figure 2).

In addition to EVs-enclosed molecules, non-EVs-enclosed molecules also play important roles though they are not our focus here. For example, cardiac macrophage miR-21 protects the heart against pressure overload-induced cardiac dysfunction by regulating the transition from fibroblasts to myofibroblasts [69]. Hypoxia stimulus induces blood macrophage transition from pro-inflammatory to pro-reparative via regulating the AMPKα2 pathway, thereby promoting myocardial repair post-MI [70].

### 3.3. Cardiomyopathy

Cardiomyopathies are a group of cardiac disorders with an unknown etiology, complex classification, and no specific treatment. Advances in the study of EVs in cardiomyopathies will provide options for precise treatment in the future. Increasing evidence shows that EVs are a potential strategy to treat cardiomyopathy. For example, EVs derived from bone marrow reduce cardiac M1 macrophages while increasing M2 macrophages by activating the JAK2-STAT6 pathway, improving cardiac function as well as attenuating myocardial cell apoptosis in an Adriamycin-induced dilated cardiomyopathy model in mice [71].

In the context of doxorubicin-induced cardiac dysfunction, there is a significant inflammatory response, cell apoptosis, and M1 polarization of macrophages that are pro-inflammatory. EVs derived from embryonic stem cells (ESC) can reverse this reaction and promote the secretion of IL-10. Furthermore, ESC-derived EVs significantly inhibit cytoplasmic vacuole formation, myofibril loss, and myocardial hypertrophy in addition to improving cardiac function [72]. In an effort to clarify the mechanism by which cardiomyopathy occurs, EVs derived from hypertrophic cardiomyocytes (HC) are being investigated. Through targeting miR-155, HC-derived EVs induce the phosphorylation of ERK, JNK, and p38, demonstrating the importance of exosomal miRNA in regulating cardiac hypertrophy [73]. In the case of other sources of cardiac dysfunction, such as air pollution, EVs containing miR-421 have the potential to reduce heart injury by suppressing ACE2, but further studies are required to determine the relation between miR-421 and macrophages [27]. A randomized controlled trial shows that cardio-sphere-derived EVs can deliver miR-4488 and improve arrhythmogenic cardiomyopathy via suppressing NF-ĸB signaling in mice, indicating indirect evidence for possible cross-talk between cardiomyocytes and macrophages [74]. What’s more, exosomal miR-34-5p has been shown to play a key role in mediating cross-talk between cardiomyocytes and macrophages in the cardiac dysfunction model [75]. Interestingly, it is not a single direction whereas is a two-sided cross-talk. The macrophage can produce EVs-enclosed toll-like receptor 9 and attenuate sepsis-induced cardiomyocyte apoptosis [76]. In addition, in sepsis-induced cardiomyopathy, MAPK signaling is involved in macrophage polarization, which regulates cardiomyocyte apoptosis [77]. Table 1 summarizes the regulation and effects of exosomal ncRNA on macrophages in different CVD.

### 3.4. Diabetic Cardiomyopathy

Diabetes is a public health burden worldwide, leading to a range of adverse cardiovascular events, including diabetic cardiomyopathy (DCM), mainly in the form of myocardial remodeling, myocardial fibrosis, diastolic dysfunction in the early stages, as well as systolic dysfunction later as the course of the disease progresses [81]. However, the pathogenesis is not fully understood and there is a lack of specific diagnostic and treatment methods. Clinical studies have found that even tight glycemic control does not improve cardiac function in diabetic patients [82]. Therefore, finding effective intervention targets and methods for diabetic cardiomyopathy, and providing a theoretical basis and promising interventions for the clinical prevention and treatment of diabetic cardiomyopathy are urgent issues that need to be addressed. Although macrophages play a critical role in chronic inflammatory cardiac remodeling in diabetic cardiomyopathy, the dysregulated balance between pro- and anti-inflammatory phenotypes contributes to excessive inflammation and cardiac injury [83]. In streptozotocin (STZ)-induced diabetic cardiomyopathy, inflammatory factors, inflammasome formation, and monocyte and macrophage infiltration are significantly elevated [84]. Several miRNAs have been identified to be associated with DCM, including miR-471-3p, miR-455, and miR-29b. According to Liu et al., inhibiting miR-471-3p decreases macrophage inflammatory polarization [85]. In db/db mouse models, Chaturvedi et al. found that miR-455 and miR-29b reduce matrix metalloproteinase 9 (MMP9) expression, therefore alleviating diabetic cardiac complications [86]. Exosomal miRNA acts as a critical role in diabetic cardiomyopathy by polarizing macrophages and this could have potential therapeutic implications. Despite few studies have been performed to investigate the mechanism of EVs-associated macrophage regulation in DCM, ncRNA seems to play a certain regulatory role as an EVs-independent way to regulate macrophage response in DCM. It has been shown that macrophages derived exosome-enclosed human antigen R could significantly increase inflammation and cardiac fibrosis [87], whereas EVs-enclosed ncRNA in regulating macrophage in DCM is still needed to elucidate. In addition, miR-471-3p is involved in contributing to M1 macrophage polarization in DCM via targeting SIRT1 signaling [85]. A recent study reveals that a reduction of lipocalin 10 in diabetic macrophages is responsible for diabetes-associated cardiac function [88].

Notably, during cardiac injury, pericardial adipose tissue can be a major source of inflammatory M1 macrophages. This means that pericardial adipose tissue could have an imminent role in the involvement of macrophages in heart injury and repair.

### 3.5. Viral Myocarditis

Myocarditis, which is an inflammatory response of the cardiac muscle characterized by cardiomyocyte necrosis or degeneration, remains one of the leading causes of heart transplantation worldwide due to the lack of a specific treatment [89]. The most common cause of myocarditis is a viral infection, and both direct reactions mediated by viruses and immune-mediated damage to cardiac tissue result in subsequent dysfunction of the heart. Activation of the immune system is necessary for eliminating the virus and restoration of myocardial tissue function, but at the same time, it may lead to necrosis, inflammation, and ventricular dysfunction [90]. Macrophages are important for the removal of debris from apoptotic cells and for the recovery of tissues following viral infection. However, activated infiltrating immune cells increase the expression of MMP, which in turn promotes immune cell recruitment and inflammation [91]. Therefore, some strategies targeting M1 macrophages may be helpful for attenuating myocarditis in certain conditions. For example, MEG3, a type of lncRNA found in polarized M2 macrophage-derived EVs, has been shown to attenuate the inflammatory response in an ulcerative colitis murine animal model [92]. Conversely, in a mouse model of viral myocarditis (VMC) induced by coxsackievirus B3 (CVB3), suppression of lncRNA MEG3 was found to reduce the polarization of M1 macrophages and induce M2 macrophage polarization, thus alleviating inflammation of the heart [93]. In the same model of CVB3-induced inflammation and myocardial damage, miR-19b-3p and its target are identified to modulate macrophage polarization [94] and upregulation of miR-192 by lncRNA AK085865ke3 promotes macrophage polarization to M2 type in VMC [95]. Notably, it is not only beneficial when inhibiting M1 macrophage in treating myocarditis. For example, it has been previously suggested that excess suppression of inflammation may affect the effectiveness of treatment [96]. Collectively, these data suggest that ncRNAs carried by different cell-derived EVs or in different microenvironments may have different polarization effects on macrophages.

## 4. Regulation of Macrophage Polarization by Immune Cell-Derived EVs and Their Role in Cardiovascular Disease

Macrophages are important regulators of the immune system, activating immune cells and promoting their proliferation to produce innate and adaptive immune responses [97]. In turn, immune cells, such as dendritic cells, neutrophils, lymphocytes, and their derived EVs are involved in regulating macrophage polarization.

The dendritic cells (DCs), the main antigen-presenting cells in the immune system, and their derived EVs (DC-EVs) share the same molecular composition as their originating cells, acting on effective targets via paracrine, endocrine, and other pathways, participating in immune responses, cell differentiation, apoptosis, proliferation, and other pathophysiological processes [98]. When compared to DCs, DC-EVs exhibit natural advantages in preservation and cell-free therapy, which may prove useful in treating cardiovascular diseases [99]. Several molecules have been identified within DC-EVs.

In addition, Treg cells can also directly produce EVs to regulate macrophage polarization. Using Treg-derived EVs to treat MI mice, it has been found not only that the EVs improve cardiac function, reduce myocardial infarction area, and inhibit apoptosis of cardiomyocytes in mice, but also they reduce the expression of M1 macrophage markers while promoting the expression of M2 macrophage markers [100].

All immune cells involved in inflammation can produce EVs, which have multiple roles in the inflammatory process and carry arachidonic acid-derived bioactive mediators that may have chemotactic effects [101]. EVs released from neutrophils can inhibit macrophage responses to LPS-induced inflammation through the MerTK signaling pathway and induce the secretion of anti-inflammatory TGF-β1 [102].

B cells can increase myocardial inflammation by directly inhibiting M2 macrophage polarization [103]. EVs derived from B cells carry MHC-II complexes, as well as co-stimulatory and adhesion molecules, and have similar antigen-presenting capabilities as the originating B cells [104]. The different types of antigens they carry may determine different types of immune responses [105]. However, few preclinical studies have examined how B cells EVs regulate macrophage polarization. Similar to B cells, T cells release EVs with heterogeneous characteristics, that are capable of targeting different kinds of immune cells and altering their function [106]. When EVs deriving from Pyruvate kinase muscle isoenzyme 2 (PKM2)-stimulated T cells were applied to macrophages in vitro, they promoted iron accumulation, lipid peroxidation, and macrophage migration [107]. In addition, EVs from activated CD4^+^ T cells can induce oxidative stress in cardiac endothelial cells by transporting MEK1/2 and ERK1/2 [108]. In monocytes, EVs derived from T cells are internalized via endogenous phosphatidylserine receptors, and lead to cholesterol accumulation [109]. It should be noted that deeper research is required to determine the effect of T cell-derived EVs on macrophage polarization.

## 5. Role of Polarized Macrophage-Derived EVs in CVD

In addition to playing a role in the regulation of CVD, macrophages are also able to mediate intercellular communication through the production of their derived EVs, which play a crucial role in the regulation of all inflammation-related diseases. M2 macrophages have a prominent role in anti-inflammatory activity, promote myocardial tissue repair, and their derived EVs are also strongly associated with cardioprotection. EVs derived from M2 macrophages can inhibit IRI, reduce NLRP3 inflammatory activity, and attenuate myocardial stunning through alleviation of oxidative stress [110], which may pave the way for developing new therapies against cardiomyocyte pyroptosis in MI. In contrast, M1 macrophages exacerbate CVD development due to their production of pro-inflammatory cytokines.

Mechanistically, miRNAs enclosed in macrophage-derived EVs mediate the cardioprotective role of M2 in ischemic heart disease. Dai et al. report that M2 macrophage-derived EVs can transfer miR-148a to attenuate IRI injury by downregulating TXNIP and inhibiting TLR4/NF-κB/NLRP3 axis [111]. Moreover, MiR-1271-5p enclosed in M2 macrophage-derived EVs can attenuate cardiomyocyte apoptosis in AMI mice and promote cardiac repair by downregulating SOX6 [112]. Further, miR-378a-3p and miR-24-3p are also effective in reducing cardiomyocyte scorching and reducing myocardial injury in mice after MI [113,114]. In reality, M2-derived EVs may not always be protective. For example, circUbe3a contained within M2 macrophage-derived EVs was shown to exacerbate the progression of myocardial fibrosis after AMI, including proliferating, migrating, and altering the phenotypic characteristics of cardiac fibroblasts through direct interaction with miR-138-5p/RhoC signaling [115]. Overall, immunocyte-derived EVs have great potential to promote angiogenesis, reduce fibrosis, and enhance cardiac function, and their function is superior to that of MSCs-derived EVs [116].

Bone marrow-derived macrophages secret EVs containing miR-99a/146b/378a, which can suppress inflammatory response by suppressing NF-κB and TNF-α signaling and further promote M2 polarization, ultimately attenuating the inflammatory response in AS [117]. Several molecules have been identified to be involved in improving AS. For example, M2 macrophage-derived EVs containing miR-221-3p promote the proliferation of endothelial cells and inhibit inflammation and apoptosis in AS [118] while miR-199a-5p reduces endothelial cell pyroptosis and mitigates AS progression via the SMARCA4/PODXL/NF-κB signaling pathway [119], providing promising strategies for treating AS. Macrophage-derived EVs carrying miR-4532 markedly impaired endothelial cell function by regulating SP1 and downstream NF-κB P65 activation [120]. In AS, MiR-503-5p enhances inflammatory cytokines and adhesion molecules released by macrophages, mediating intercellular communication with endothelial cells and vascular SMCs [121]. MiR-186-5p and miR-106a-3p contained in oxidized low-density lipoprotein (Ox-LDL)-exposed macrophage-derived EVs can enhance the viability of SMC and inhibit their apoptosis [122,123]. LDL-induced macrophage-EVs target JAZF1 via miR-19b-3p to accelerate SMC migration and proliferation, thereby promoting AS progress [124]. Taken together, these studies show that there is potential for novel therapeutic approaches for AS patients based on miRNAs encapsulated in macrophage-derived EVs.

However, EVs derived from M1 macrophages can impair cardiac function, exacerbate myocardial fibrosis and promote apoptosis. MiRNAs (miR-185-3p, miR-16-5p) enclosed in M1 macrophage-derived EVs exacerbate the development of CVD [125,126]. Pro-inflammatory M1 macrophages release pro-inflammatory EVs after MI, and their high expression of pro-inflammatory miRNAs, such as miR-155, can exert anti-angiogenic effects and accelerate MI injury [127]. In addition, miR-155 can act as a paracrine regulator of fibroblast inflammation and proliferation [57], and promote cardiomyocyte pyroptosis and cardiomyocyte hypertrophy in uremic cardiomyopathy [128]. Thus, inhibitors of miR-155 have great potential to be used as a therapeutic agent to reduce adverse events associated with AMI and to attenuate uremic cardiomyopathy. The downregulation of miR-21-5p in MI-macrophage-derived EVs inhibits ventricular remodeling after MI by suppressing tissue inhibitor of metalloproteinase 3 (TIMP3) [129]. EVs can also increase the expression of CDC42 and activate the MEK/ERK pathway through their lncRNA such as MALAT1, which binds to miR-25-3p and inhibits angiogenesis and myocardial regeneration after MI, ultimately hindering the repair and regeneration process of the heart [130]. These studies provide new insights into the role of macrophages and their derived EVs in the treatment of CVD.

In addition to secreting EVs, macrophages have gradually attracted researchers’ attention for their role in maintaining tissue homeostasis through efferocytosis [131]. In chronic inflammatory CVD, efferocytosis is defective, allowing a gradual accumulation of apoptotic cells that undergo necrosis, leading to a pathological inflammatory response [132]. Following MI, cardiac-resident macrophages are responsible for clearing apoptotic cardiomyocytes, a process necessary for inflammation regression and tissue repair. It has been found that macrophage efferocytosis induces the production of vascular endothelial growth factor C and promotes the formation of cardiac lymphatic vessels in order to reduce inflammatory response and cardiac injury after MI [133]. Impaired clearance of dead cells from atherosclerotic plaques by macrophages leads to the formation of atherosclerotic plaques and acute coronary artery disease [134,135]. Kasikara et al. showed that the defective PHACTR1 gene in macrophages inhibits dephosphorylation of the myosin light chain which is required for apoptotic vesicles, thereby impairing efferocytosis and promoting atherosclerotic plaque necrosis [136]. Aldehyde dehydrogenase 2 (ALDH2) rs671 mutation is a threat factor for the progression of AS that confers a poor prognosis [137]. ALDH2 can directly interact with Rac2 to regulate the ubiquitination of Rac2, thereby promoting macrophage efferocytosis and reducing AS [138]. Investigations of macrophage efferocytosis could provide potential therapeutic strategies for AS.

## 6. A Role for EVs in the Treatment of CVD by Acting on Macrophages

### 6.1. Stem Cells-Derived EVs

The repair and regenerative capacity of stem cells hold great promise in CVD treatment, but their immunogenicity, storage, and stability limit their use in clinical settings. As a cell-free nanotherapeutic tool that avoids these drawbacks, EVs are gradually attracting the attention of researchers.

**Figure 3:**
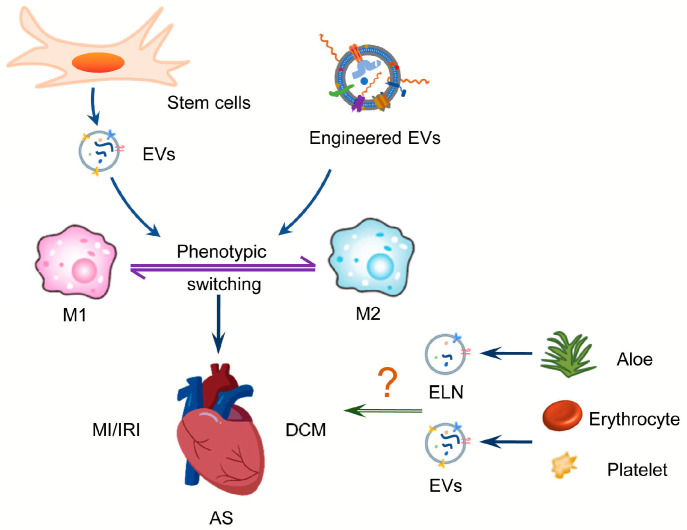
A role for EVs in the treatment of CVD by acting on macrophages. Stem cell-derived EVs or engineered EVs can protect the heart against MI/IRI, DCM, and AS by regulating macrophage polarization. In addition, the effect of nanoparticles derived from plants, erythrocytes, and platelets on macrophage polarization still needs further investigation. Abbreviations: MI/IRI: myocardial infarction/ischemia reperfusion injury; DCM: diabetic cardiomyopathy; AS: atherosclerosis; ELN: exosome-like nanovesicles.

Several studies have demonstrated that MSC-derived EVs alleviate CVD such as MI and IRI by regulating macrophage polarization [59,60,63] (Figure 3). Sun et al. show that intravenous injection of MSCs-derived EVs improves the cardiac inflammatory microenvironment in a mouse model with DCM through regulation of macrophage polarization [71], which may offer hope for the clinical management of DCM. According to Monguió-Tortajada et al., local delivery of MSCs-derived EVs to an AMI pig model demonstrated preclinical efficacy [139], indicating the great potential of MSCs-EVs in regulating cardiac repair after MI. de Almeida Oliveira et al. demonstrate that miR-196a-5p and miR-425-5p in adipose tissue-derived EVs prevent ischemia-induced mitochondrial dysfunction and oxidative stress in cardiomyocytes, increase angiogenesis, and promote macrophage polarization toward the anti-inflammatory M2 type [140]. In addition, adipose tissue stem cell-derived EVs restore endothelial dysfunction caused by AS [141], which confirms its potential as a therapeutic tool.

### 6.2. Engineered EVs

In recent years, researchers have created EV-based gene therapies by either modifying protoplasts or directly engineering modified EVs (Figure 3). Stamatikos et al. constructed a helper-dependent adenovirus (HDAd) that expressed an antagomiR directed at miR-33a-5p. Macrophages or SMC exposed to EVs containing anti-miR-33a-5p were shown to slow down the development of AS [51]. Encapsulating engineered IL-10 mRNA into EVs can be effectively delivered to macrophages in atherosclerotic plaques of ApoE^−/−^ mice and reduce side effects due to immunosuppression [142]. Engineered M2 macrophage-derived EVs exhibit excellent anti-inflammatory roles through surface-bonded chemokine receptors and anti-inflammatory cytokines, leading to the alleviation of AS [143]. Recently, a two-step approach has been developed based on CD-47 modification on miR-21 enriched MSC-EVs, which results in a significant reduction of EV clearance and efficient uptake by cardiomyocytes, thereby inhibiting cell apoptosis, reducing inflammation, and improving cardiac function [144]. As a result of this new approach, EVs are more likely to be retained, which may prove to be a promising therapeutic strategy for improving CVD treatments.

### 6.3. Important but Blank Space for Macrophage Regulation in CVD

#### 6.3.1. Plant-Derived Exosome-like Nanovesicles

Plant cells are capable of releasing vesicles, namely exosome-like nanovesicles (ELN), which have been proven to be effective in attenuating pathological processes [145]. Plant-derived EVs also reduce the expression of inflammatory cytokines and decrease the activation of NF-κB in LPS-stimulated mouse macrophages, thus exerting anti-inflammatory effects [146]. In addition, EVs derived from Aloe vera exhibit antioxidant properties and promote wound healing [147,148], making them a potential drug for cardiac repair in IRI. Other plant-derived EVs, such as grapefruit and ginger, are gradually attracting research attention as a new approach to cell-free therapy in intestinal diseases [149,150], however, their application in CVD needs to be further explored (Figure 3).

#### 6.3.2. Microparticles Derived from Erythrocytes and Platelets

Accumulating evidence indicates that EVs derived from platelets and erythrocytes play an important role in coagulation and in cardiovascular diseases such as hypertension and AS [151] (Figure 3). Circulating particles derived from ischemic preconditioning (IPC-MV), including platelet- and erythrocyte-derived particles, are significantly increased and can protect the heart from IRI by attenuating endoplasmic reticulum stress [152], which provides new insights into the clinical treatment of IRI by IPC-MV. In fact, Giannopoulos et al. examined circulating particles in blood after angioplasty (PCI) in AMI patients and observed elevated erythrocyte particles, whose concentration was positively connected with adverse clinical events [153]. Using erythrocyte-derived EVs (REVs) and therapeutic siRNAs, Tang et al. target Kim-1 in mouse kidneys and deliver siRNAs to alleviate renal ischemia and reperfusion damage [154]. It has been demonstrated that erythroid-derived EVs can promote the phenotypic transformation of pro-inflammatory macrophages and exacerbate the inflammatory response. The underlying mechanism may involve EV-mediated upregulation of TLR4-MyD88-NF-B-MAPK activity [155]. The action of erythroid-derived EVs on macrophages may be a new direction to attenuate the inflammatory response in CVD.

Notably, some of the ncRNAs summarized in this study have been suggested to be used for disease diagnosis and therapy prognosis. Here we summarize some common and well-studied ncRNAs. For example, miR-92a is found to be associated with the development of DCM, acute MI, CAD, and AS and is a potential diagnostic marker of these diseases [156,157,158,159]. miR-21-5p can be a great candidate for indicating acute MI [160] and acute viral myocarditis [161] etc. In addition, miR-181b is identified as a potential circulating biomarker for heart failure-associated inflammation [162] and diabetic cardiomyopathy [163]. Therefore, studying the mechanism of CVD upon EVs-enclosed ncRNA may help to identify new circulating biomarkers for the diagnosis and prognosis of CVD.

## 7. Limitations

Actually, to clearly describe the regulation of macrophage polarization and its role in CVD, we use the M1/M2 paradigm in the present study. However, it should be noted that M1/M2 classification has its specific applicable limitation including its in vitro construction, which relies on some stimulations by defined factors [164]. Therefore, we may need a more appropriate system to define macrophage subtypes although we use M1/M2 paradigm in the present study. On the other hand, we do not distinguish between subtypes of M2 macrophages here. The previous study demonstrates that M2 macrophage can be induced for different subgroups with specific markers, including IL-4/IL-13 induced M2a, immune complex/IL-β/TLR induced M2b, IL-10/TGF-β/glucocorticoids induced M2c (prominent) and IL-6/adenosine A2A receptor-induced M2d. In addition to M1 and M2 macrophages, other subtypes such as M4 and M(Hb) are identified in atherosclerotic plaques [165]. MSC-derived secretomes can promote macrophages to polarize to M2b/M2c-like subsets, thereby suppressing inflammation [166,167]. Especially, M2b macrophages play both pathogenic and protective roles in various diseases, which can be a promising therapeutical strategy for inflammatory diseases [168]. Nevertheless, the role and mechanism of EVs-enclosed ncRNAs in regulating M2 subcategories in CVD are still needed to be elucidated.

## 8. Conclusions

EVs, messengers for cell-to-cell communication, are associated with macrophage polarization, inflammatory response, and other pathological processes, and therefore play a critical role in CVD progression. Exosomal ncRNA can promote the activation of M2 macrophages, produce anti-inflammatory cytokines, and mitigate pathological processes by regulating macrophage M1 subtype changes. An in-depth investigation of the role that EVs play in different pathological processes may provide a new direction for the treatment of CVD. Further, macrophages, one of the most important components of the immune system, can communicate with a variety of cells via EVs. Undoubtedly, there is a wide range of therapeutic possibilities for DC and Treg cell-derived EVs in the field of CVD. Furthermore, EVs derived from stem cells, such as MSCs, have gradually gained recognition as a new research direction and therapeutic target for CVD. However, further research is necessary to examine the mechanisms underlying the effects of ncRNA encapsulated in EVs, especially circRNA and lncRNA, on macrophage phenotypic changes. EVs have also been extensively studied as gene therapy vectors for the treatment of CVD in the last few years; however, how to precisely target the lesion site needs further investigation in future studies.

## Figures and Tables

**Table 1:** Effects and regulation of exosomal ncRNA on macrophage in CVD.

Model	Cell of Origin	Contents	Effects	Target Gene/Single Pathway	Reference
AS	endothelial cells	miR-92a	promote M1 macrophage polarization	suppressed the expression of the target gene KLF4	[45]
AS	endothelial cells	miR-33	promote pro-inflammatory macrophage activation	targeting to NR4A	[46]
AS	perivascular adipose tissue	miR-382-5p	reduce macrophage foam cell formation	BMP4-PPARγ-ABCA1/ABCG1 pathways	[48]
AS	adipose tissue	/	induce M1 phenotype and proinflammatory cytokine release	increased phosphorylation of NF-κB-p65	[47]
AS	adipose tissue-derived MSCs	/	increase the expression of M2 markers	inhibiting MAPK, NF-κB pathways and activating the STAT3 pathway	[49]
AS	mesenchymal stem cells	miR-let7	promote M2 macrophage polarization	HMGA2/NF-κB and IGF2BP1/PTEN pathway	[50]
AS	mesenchymal stem cells	miR-21a-5p	promote M2 macrophage polarization	Inhibit KLF6 and ERK1/2 single pathway	[31]
MI/MIR	cardiomyocyte	miR-155-5p	facilitate M1 polarization with increased expression of inflammatory cytokines	activating JAK2/STAT1 pathway	[54]
MI/MIR	mesenchymal stem cells	miR-182	modify the polarization of M1 macrophages to M2 macrophages	TLR4 as a downstream target	[59]
MI/MIR	mesenchymal stem cells	miR-21-5p	promote M2 macrophage polarization	/	[32]
MI/MIR	adipose tissue-derived MSCs	/	promote M2 macrophage polarization	S1P/SK1/S1PR1 pathway	[60]
MI/MIR	cardiac progenitor cell	/	reduce M1 macrophages and increase M2 macrophages	/	[78]
MI/MIR	Cardio-sphere derived cell	/	increase M1 macrophage polarization	arginase 1 upregulation	[67]
MI/MIR	Cardio-sphere derived cell	miR-181b	reduce CD68+ macrophage and modify the polarization state of macrophage	targeting to PKCδ	[79]
MI/MIR	Cardio-sphere derived cell	Y RNA	Induce the secretion of IL-10 in macrophages	/	[68]
DCM	mesenchymal stem cells	/	reduce M1 macrophages and increase M2 macrophages	JAK2-STAT6 pathway	[71]
HCM	Cardio-sphere derived cell	YF1	decrease proinflammatory monocytes	reverse hypertrophic and fibrotic signaling pathways	[80]
HCM	hypertrophic cardiomyocytes	miR-155	increased expression of proinflammatory cytokines	increased phosphorylation of ERK, JNK and p38	[73]
DIC	embryonic stem cell	/	increase M2 macrophages and IL-10	/	[72]

AS: atherosclerosis; MI/MIR: myocardial infarction/ myocardial ischemia reperfusion injury; DCM: dilated cardiomyopathy; HCM: hypertrophic cardiomyopathy; DIC: Doxorubicin-Induced cardiomyopathy.

## Data Availability

Not applicable.
